# Supplementary material for: EEG-based vibrotactile evoked brain-computer interfaces system: A systematic review
Source: PLoS One. 2022 Jun 3;17(6):e0269001. doi: 10.1371/journal.pone.0269001 (PMC9165854; doi:10.1371/journal.pone.0269001)
Supplement: S1 Appendix — (DOCX) [file pone.0269001.s002.docx]

**Searching queries for each database**

- **Pubmed**

(electroencephalography OR electroencephalographies OR EEG OR eeg OR "brain electrical activity" OR "electric encephalogram" OR "electro encephalogram" OR electroencephalogram OR "brain wave" OR biofeedback OR neurofeedback OR ("electroencephalography"[MeSH Terms]))

AND

(BCI OR "brain-computer interface" OR "brain-machine interface" OR "direct neural interface" OR "noninvasive brain-computer interface" OR "noninvasive brain computer interface" OR ("brain-computer interfaces"[MeSH Terms]))

AND

(vibration OR vibrational OR vibrotactile OR vibrations OR vibrate OR vibrated OR vibrates OR vibrating OR vibrator OR vibrators OR "vibration sense" OR tactual OR "sense of touch" OR "touch stimulus" OR tactility OR "tactile stimulation" OR tactile OR touch OR haptic OR (vibration[MeSH Terms]))

- **Embase**

(electroencephalography OR electroencephalographies OR EEG OR eeg OR "brain electrical activity" OR "electric encephalogram" OR "electro encephalogram" OR electroencephalogram OR "brain wave" OR biofeedback OR neurofeedback OR "electroencephalography"/exp)

AND

(BCI OR "brain-computer interface" OR "brain-machine interface" OR "direct neural interface" OR "noninvasive brain-computer interface" OR "noninvasive brain computer interface" OR "noninvasive brain-computer interface"/exp)

AND

(vibration OR vibrational OR vibrotactile OR vibrations OR vibrate OR vibrated OR vibrates OR vibrating OR vibrator OR vibrators OR "vibration sense" OR tactual OR "sense of touch" OR "touch stimulus" OR tactility OR "tactile stimulation" OR tactile OR touch OR haptic OR " vibration"/exp)

- **Web of Science**

(ALL= electroencephalography OR ALL= electroencephalographies OR ALL= EEG OR ALL= eeg OR ALL="brain electrical activity" OR ALL="electric encephalogram" OR ALL= "electro encephalogram" OR ALL= electroencephalogram OR ALL= "brain wave” OR ALL= biofeedback OR ALL= neurofeedback)

AND

(ALL=BCI OR ALL="brain-computer interface" OR ALL="brain-machine interface" OR ALL="direct neural interface" OR ALL="noninvasive brain-computer interface" OR ALL="noninvasive brain computer interface")

AND

(ALL= vibration OR ALL= vibrational OR ALL= vibrotactile OR ALL=vibrations OR ALL=vibrate OR ALL=vibrated OR ALL=vibrates OR ALL=vibrating OR ALL=vibrator OR ALL=vibrators OR ALL= "vibration sense" OR ALL=tactual OR ALL="sense of touch" OR ALL="touch stimulus" OR ALL=tactility OR ALL= "tactile stimulation" OR ALL= tactile OR ALL= touch OR ALL= haptic)

- **IEEE Explore**

(electroencephalography OR electroencephalographies OR EEG OR eeg OR "brain electrical activity" OR "electric encephalogram" OR "electro encephalogram" OR electroencephalogram OR "brain wave" OR biofeedback OR neurofeedback OR ("Mesh_Terms":electroencephalography))

AND

(BCI OR "brain-computer interface" OR "brain-machine interface" OR "direct neural interface" OR "noninvasive brain-computer interface" OR "noninvasive brain computer interface" OR ("Mesh_Terms":brain-computer interfaces))

AND

(vibration OR vibrational OR vibrotactile OR vibrations OR vibrate OR vibrated OR vibrates OR vibrating OR vibrator OR vibrators OR "vibration sense" OR tactual OR "sense of touch" OR "touch stimulus" OR tactility OR "tactile stimulation" OR tactile OR touch OR haptic OR ("Mesh_Terms":vibration))

- **PsycINFO**

(electroencephalography OR electroencephalographies OR EEG OR eeg OR "brain electrical activity" OR "electric encephalogram" OR "electro encephalogram" OR electroencephalogram OR "brain wave" OR biofeedback OR neurofeedback OR MAINSUBJECT.EXACT("Electroencephalography")) AND (BCI OR "brain-computer interface" OR "brain-machine interface" OR "direct neural interface" OR "noninvasive brain-computer interface" OR "noninvasive brain computer interface") AND (vibration OR vibrational OR vibrotactile OR vibrations OR vibrate OR vibrated OR vibrates OR vibrating OR vibrator OR vibrators OR "vibration sense" OR tactual OR "sense of touch" OR "touch stimulus" OR tactility OR "tactile stimulation" OR tactile OR touch OR haptic OR MAINSUBJECT.EXACT("Vibration"))
